# Biomarkers of Acute Kidney Injury

**DOI:** 10.1155/2011/328120

**Published:** 2011-10-29

**Authors:** Jeffrey C. Sirota, Jelena Klawitter, Charles L. Edelstein

**Affiliations:** ^1^Division of Renal Diseases & Hypertension, University of Colorado Health Sciences Center, Mail Stop C-281, 12700 East 19th Avenue, Aurora, CO 80045, USA; ^2^Department of Anesthesiology, University of Colorado Health Sciences Center, Mail Stop C-281, 12700 East 19th Avenue, Aurora, CO 80045, USA

## Abstract

Acute kidney injury (AKI) is a common problem in both the inpatient and outpatient setting and often results from drug toxicities. Traditional methods of identifying AKI, through measurement of blood urea nitrogen and serum creatinine, are problematic in that they are slow to detect decreases in glomerular filtration rate (GFR) and are influenced by a variety of factors that are not related to GFR changes. The problems inherent in a creatinine-based diagnosis of AKI have impeded the development of proper therapeutics in AKI and posed problems in evaluating nephrotoxicity of drugs and other chemical exposures. In recent years, a number of new biomarkers of AKI with more favorable test characteristics than creatinine have been identified and studied in a variety of experimental and clinical settings. This review will consider the most well-established biomarkers and appraise the literature, with particular attention given to the use of biomarkers in identifying toxin-mediated AKI.

## 1. Introduction

AKI, defined as a rapid decline in GFR, is a common problem, and the incidence has been increasing significantly, particularly in the hospital setting. Recent estimates have suggested that AKI accounts for 1% of all hospital admissions, complicates 7% of hospitalizations, and is present in up to 20% of critically ill patients [1]. Despite significant advances in both critical care and nephrology, the mortality rate of hospitalized patients with AKI has remained relatively unchanged at around 50% over the past few decades [2]. A wide variety of etiologies underlies community-acquired AKI, while ischemia, sepsis, and toxins (including medications) are the most common etiologies in hospitalized patients.

Criteria for the diagnosis of AKI rely heavily on measurements of serum creatinine, a common practice for estimating renal function for over sixty years. Creatinine is a 113 Dalton molecule that is derived from phosphocreatinne, which in turn is a product of creatine metabolism after creatine's release from muscle. Creatinine is freely filtered by the glomerulus and excreted thereafter without significant metabolism or reabsorption by the kidney [3]. These properties have made serum creatinine level a useful surrogate for kidney function, and the reciprocal relationship between serum creatinine and GFR has been well described [4]. A variety of equations (e.g., Cockcroft-Gault, MDRD, and CKD-EPI) have been developed to estimate GFR from a measured serum creatinine level.

Despite its widespread use, however, serum creatinine has significant limitations as a tool for assessing GFR. First of all, a variety of nonrenal factors influence creatinine production rate so that creatinine production is variable not only from one patient to the next, but also within the same patient placed under different conditions. Most notably, age, gender, muscle mass, diet (particularly protein intake), and nutritional status are major determinants of creatinine production [5]. While some equations for eGFR account for some of these variables (such as age and gender in the MDRD equation), muscle mass and nutritional considerations are not reflected by these equations.

In addition to the variability of creatinine production, tubular secretion of creatinine can also vary and cause error in creatinine-derived estimates of GFR. Under normal conditions, tubular secretion of creatinine accounts for roughly 10% of creatinine clearance, but this secretion is inhibited by certain medications such as trimethoprim and cimetidine, leading to elevations in serum creatinine that do not reflect true decrements in GFR [6]. In addition, because it is measured as a concentration, serum creatinine is influenced by its volume of distribution, which can be significantly altered by volume overload (a condition commonly present in both acute and chronic renal insufficiency) [7].

While the influence of non-renal factors makes serum creatinine problematic for estimating GFR under normal circumstances, a variety of other problems exist in relying on serum creatinine for diagnosing AKI. Most importantly, the temporal relationship between decreases in GFR and resultant elevations in serum creatinine precludes early recognition of significant GFR loss. It has long been known that substantial loss of GFR may not manifest with elevations in serum creatinine for several days [8]. In addition, during non-steady-state conditions, creatinine-based estimates of GFR are inaccurate, making assessment of true renal function difficult. Finally, significant impairment in GFR increases the proportion of creatinine clearance that can be ascribed to tubular secretion, which can result in a substantial overestimate of GFR once the true GFR falls below 15 mL/min [9].

These myriad problems with creatinine limit both clinical practice and the development of new therapeutics in AKI. To improve recognition and intervention in AKI, clinicians need tools that are not influenced by other clinical parameters or patient characteristics and that can identify losses in GFR soon after they occur. An ideal biomarker of AKI would be a substance that the kidney releases immediately in response to injury, and that can be detected in the blood or urine without significant metabolism. This biomarker would be highly sensitive and specific for injury to the kidney, whether or not that injury influences total GFR. Biomarkers with similar favorable characteristics have been employed in other areas of clinical practice, such as troponin measurement in the identification of myocardial injury.

A variety of new protein-based biomarkers of kidney injury have been identified and may augment the traditional evaluation of kidney function, which has primarily relied on measurement of small molecules such as creatinine and urea [10, 11]. In addition, decades of animal research have explored the use of metabolic profiling for the monitoring of kidney function and specific and sensitive detection of injury [12–15]. Combinatorial metabolite markers have shown promise and are attractive due to their superior stability compared to most proteins and the availability of validated and quantitative assays. However, at this point in the development of metabolic markers, the aforementioned protein biomarkers of kidney function are closer to making an impact on clinical practice than novel metabolite marker strategies.

While there has been significant progress in identifying clinical biomarkers of AKI, the field is still developing. This area of research and development is highly important, as improved detection of AKI through the use of biomarkers might lead to improved patient care by allowing more careful avoidance of nephrotoxins, appropriate modification of drug dosing, further attention to fluid status, and possibly therapeutic interventions that have thus far failed to show benefit due to late detection through creatinine-based monitoring. Toxin-mediated AKI might be particularly amenable to biomarker detection given the direct tubular injury imparted by various nephrotoxins. The remainder of this review will consider the most prominent biomarkers currently under investigation, with particular attention to toxin-mediated AKI.  Table 1 provides references to the most prominent studies of AKI biomarkers that will be described below.

## 2. Overview of Biomarkers in Nontoxic AKI

### 2.1. Neutrophil Gelatinase-Associated Lipocalin (NGAL)

NGAL, a 21 kD a member of the lipocalin superfamily of proteins, is expressed by immune cells, hepatocytes, and renal tubular epithelial cells [52]. It was first identified as a potential marker for AKI in 2003 through a transcriptome-wide search for genes upregulated after renal ischemia [53]. This potential was tested in a series of animal studies that demonstrated early postischemic upregulation of NGAL transcription and translation, particularly in proximal tubular cells. In vitro studies with cultured human proximal tubular epithelia confirmed this molecule's ability to detect injury, as in vitro injury resulted in marked NGAL upregulation. Currently, NGAL levels are measured using either enzyme-linked immunosorbent assay (ELISA) or the Architect assay (Abbott Laboratories).

The role of NGAL as a biomarker of human AKI has been established through a variety of studies in the settings of critical illness and postcardiopulmonary bypass (CPB), with the earliest reports coming from the pediatric literature. In 2005, a study of 71 children undergoing CPB was reported. In the 20 patients who developed AKI by creatinine criteria, both serum and urine NGAL levels rose significantly within 2 hours of surgery (mean urine NGAL rose from 1.6 ug/L to 147 ug/L while serum NGAL rose from 3.2 ug/L to 61 ug/L). In contrast, creatinine elevations were not appreciated for at least 1 day following the surgery. Urinary NGAL at 2 hours was used to define a receiver-operating characteristic (ROC) curve that had an area under the curve (AUC) of 0.998, and, with a cutoff of 50 mcg/L, its sensitivity for AKI was 1.00, and specificity was 0.98 [16]. A follow-up study in 2007 examined 120 children undergoing CPB and showed similarly excellent sensitivity and specificity for the diagnosis of AKI using 2-hour plasma NGAL levels. In addition, the 2-hour postoperative NGAL levels correlated with duration of AKI and length of hospital stay, while the 12-hour NGAL level correlated strongly with mortality [17]. 

As for adults, a study in 2006 of 81 patients undergoing cardiac surgery found that NGAL levels were four-fold higher in AKI patients as early as one hour postoperatively when compared to those without AKI [20]. A similar study of 100 adults undergoing cardiac surgery showed that serum NGAL was a reliable predictor of AKI and correlated with the duration and severity of AKI [21–23]. Lastly, a study of 50 patients undergoing CPB established ROC curves for AKI prediction with AUC's of 0.80 for plasma NGAL and 0.96 for urinary NGAL [24].

In the critical care arena, NGAL's predictive value has been studied most extensively in children. A study examining urinary NGAL in 140 mechanically ventilated children found that NGAL levels increased substantially two days before a significant creatinine rise, and the degree of early NGAL elevation correlated with the severity of the AKI [31]. The rise in NGAL was six-fold higher than in controls. A subsequent study of 143 critically ill children showed significantly higher NGAL levels in patients with septic shock compared to those with the systemic inflammatory response syndrome, and both of these critically ill categories had much higher NGAL levels than healthy controls. In addition, the degree of serum NGAL elevation seen during the first intensive care unit day was significantly greater in patients who developed AKI compared to those who did not (median serum NGAL was 355 ng/mL in children with AKI as opposed to 186 ng/mL in those without) [32].

As for critically ill adults, a 2010 study evaluated 88 intensive care unit (ICU) patients and found that an NGAL level of ≥155 nmol/L predicted AKI with 82% sensitivity and 97% specificity [35]. A more recent study of 45 patients admitted with septic shock has found that urine NGAL level 12 hours before AKI diagnosis was a good predictor of AKI, with an area under the receiver-operating characteristic (ROC) curve of 0.86 [36]. While plasma NGAL in this study did not perform as well, a contemporaneous study described 301 patients admitted to an ICU in whom plasma NGAL level was a good predictor of AKI development within the next 48 hours (area under ROC curve was 0.78), with good correlation between peak NGAL levels and AKI severity [37].

NGAL has also been used in a few other clinical settings. A recent study has reported the use of NGAL in 119 patients admitted with acute heart failure, with the observation that NGAL levels at admission were higher in the 11.8% of patients who developed cardiorenal syndrome within 2-3 days of hospitalization. The area under the ROC curve for diagnosis of AKI was 0.93, and, using a cutoff of 170 ng/L, NGAL predicted the subsequent development of cardiorenal syndrome with 100% sensitivity and 86.7% specificity [54]. NGAL has also been successfully used to identify AKI in patients following orthotopic liver transplant (OLT). In a study of 95 patients undergoing OLT, plasma NGAL had an area under ROC curve of 0.87, and a composite of APACHE II score above 13 and plasma NGAL above 258 was a powerful predictor of severe postoperative AKI [55].

Interestingly, NGAL measurements at the time of hospital or ICU admission have been shown to be useful predictors of AKI in hospitalized patients. In a 2008 study, 635 adult patients admitted to a hospital each had a single urine NGAL measurement made upon arrival to the emergency department, and NGAL levels were shown not only to correlate with the development of AKI but also predict a composite endpoint of nephrology consultation, dialysis, ICU admission, or mortality with an odds ratio of 24.71 [56]. Another study of 31 patients measured urine NGAL levels in trauma patients at admission, and, with a cutoff of 25 ng/mL, urine NGAL had a sensitivity of 0.91 and specificity of 0.95 in predicting AKI [57]. For patients admitted to the ICU, NGAL can be a powerful predictor of AKI severity, as recently demonstrated in a study of 632 consecutive patients admitted to an ICU in whom both plasma and urine NGAL levels measured at admission were associated with AKI severity, and adding NGAL to a prediction model for development of AKI significantly improved the model's accuracy [58].

There is some evidence that urinary NGAL can be a useful tool in discerning the cause of AKI as well. In a recent study, 145 hospitalized patients with AKI have been classified into different categories based on the clinical cause of their AKI, and urinary NGAL levels were measured. Urinary NGAL was able to discriminate between intrinsic and prerenal causes with a ROC curve AUC of 0.87. In addition, urinary NGAL levels above 104 *μ*g/L indicated intrinsic AKI (likelihood ratio 5.97) while NGAL levels below 47 *μ*g/L made intrinsic AKI unlikely (likelihood ratio 0.2). Using biomarkers to distinguish the etiology of AKI may be of particular importance in hospitalized patients with multiple potential causes of AKI (e.g., septic patients on potentially nephrotoxic antibiotics) [59].

### 2.2. Interleukin-18 (IL-18)

Like NGAL, IL-18 plays a role in the immune system. IL-18 is a widely expressed proinflammatory cytokine weighing 18 kDa. In addition to its involvement in the immune response, this cytokine has been identified as a mediator of ischemic injury in the heart, brain, and kidney. Animal studies first explored the role of IL-18 in ischemic AKI. First, inhibiting IL-18 in mice has been shown to decrease the severity of ischemic AKI [60]. Further animal studies demonstrated significant increases in IL-18 in whole kidney after experimentally induced AKI, and a series of additional studies using transgenic models or IL-18 neutralizing antiserum have confirmed IL-18's key role in ischemic AKI [61–63]. Studies of isolated mouse proximal tubules have demonstrated increased IL-18 levels in the setting of hypoxia, and mice with ischemic AKI were found to have elevated urinary levels of IL-18 [64].

These findings in animal models have lead to extensive research on IL-18 as a biomarker of AKI in humans. IL-18 levels are typically measured through ELISA or a specific assay developed for their detection (Architect assay, Abbott Laboratories). A 2004 study first examined this possibility by measuring urinary IL-18 levels in 72 patients, 22 with AKI (14 with acute tubular necrosis [ATN], 8 prerenal), 5 with urinary tract infection, 8 with chronic kidney disease, 4 with nephrotic syndrome, 22 with renal transplant, and 11 healthy controls [65]. Among the nontransplant patients, those with ATN had significantly higher IL-18 levels than all others. In addition, transplant recipients who developed delayed graft function had significantly higher urine IL-18 levels than those with prompt graft function.

Subsequent studies examined IL-18 in other clinical situations. The predictive value of elevated IL-18 levels in critically ill patients was assessed in a nested case-control study within the Acute Respiratory Distress Syndrome Network trial (median urine IL-18 was 104 pg/mL at 24 hours in the AKI group versus 0 pg/mL in the group without AKI) [66]. Banked urine samples were assayed for IL-18 levels in 52 patients with AKI and 86 patients with no AKI. IL-18 was found to be significantly elevated up to 48 hours before the creatinine-defined occurrence of AKI. In addition, elevated IL-18 levels were found to be an independent predictor of death. A more recent study in the critical care setting has involved 451 patients, 86 of whom developed AKI. Urinary IL-18 was used to construct a ROC curve, and while the AUC was only 0.62, it was found that elevated urinary IL-18 was independently predictive of a composite outcome of death or acute dialysis within 28 days (with an odds ratio of 1.86) [67].

The predictive ability of urinary IL-18 has also been demonstrated in critically ill children in 103 pediatric patients with renal impairment and 34 controls with normal renal function, with renal failure patients manifesting a >3 fold increase in urinary IL-18 [33]. As in the adult population, urine IL-18 levels rose prior to the rise in creatinine in patients with AKI, and elevated levels were independently associated with mortality. Further, the degree of urine IL-18 elevation predicted the severity of AKI.

As in critically ill patients, AKI is also common after CPB, both in children and in adults. Comparing 20 children who developed AKI after CPB with 35 controls whose renal function remained normal, it was found that elevated IL-18 is an early predictor of AKI in this setting (with a >15-fold increase at 4 hours in AKI patients versus non-AKI patients) [18]. In adults undergoing CPB, the early data on urinary IL-18 are less clear; one study has demonstrated good predictive value for urinary IL-18 [25] while a larger study did not [26].

The most recent data on AKI following cardiac surgery comes from the Translational Research Investigating Biomarker Endpoints for Acute Kidney Injury (TRIBE-AKI) study. The pediatric part of this study measured preoperative and postoperative urine IL-18 and NGAL as well as plasma NGAL in 311 children undergoing surgery for congenital heart disease. 17% of the patients reached the primary outcome of severe AKI, defined as dialysis requirement or doubling of serum creatinine. The highest quintiles of urine IL-18 and urine NGAL were strongly associated with AKI risk (adjusted odds ratios of 6.9 and 4.1). Elevated urinary biomarkers were associated with longer ICU stay, longer hospitalization, and longer mechanical ventilation. The AUC's for the urine IL-18 ROC curve and the urine NGAL ROC curve were 0.72 and 0.71, respectively [19].

The TRIBE-AKI study group also examined these biomarkers in adults following cardiac surgery. 1219 patients undergoing cardiac surgery had urine IL-18 and NGAL as well as plasma NGAL measured prior to surgery and for five postoperative days. Multivariate analysis revealed that the highest quintiles of urine IL-18 and plasma NGAL at six hours were strongly associated with risk of AKI (adjusted odds ratios of 6.8 and 5, resp.). Higher biomarker levels were also associated with longer hospitalization, longer length of ICU stay, higher risk of dialysis, and death [68]. 

### 2.3. Kidney Injury Molecule-1 (KIM-1)

KIM-1, a type 1 transmembrane protein with an immunoglobulin and mucin domain, was first described in 1998 [69]. In normal kidney tissue, it is expressed at a low level, but, following ischemic injury, it is dramatically upregulated in regenerating proximal tubules. Animal studies have shown that levels of urinary KIM-1 increase in models of ischemic AKI, sometimes without concomitant blood urea nitrogen or creatinine elevation [70, 71]. KIM-1 levels are measured using ELISA.

KIM-1's use as a biomarker of AKI in humans was suggested in a 2002 study that described urinary KIM-1 levels in patients with AKI. In 7 patients with ischemic acute tubular necrosis (ATN), mean KIM-1 levels were significantly higher than in 16 patients with other forms of AKI (2.92 ng/mL versus 0.63 ng/mL) [72]. This study also described immunohistochemical evaluation of six patients with proven ATN; all tissue samples showed extensive KIM-1 expression in proximal tubule cells.

A 2009 report described biomarker measurements from 90 adult cardiac surgery patients, 36 of whom developed AKI [27]. A ROC curve for urinary KIM-1 drawn immediately after surgery had an AUC of 0.68, which was better than the value for NGAL. This study also demonstrated that combining multiple AKI biomarkers improved the overall predictive value. A more recent study of 123 adults undergoing cardiac surgery has shown that preoperative KIM-1 levels were able to predict the development of AKI [28].

Another study published in 2007 described 201 hospitalized patients with AKI and demonstrated a correlation between urinary KIM-1 levels and Acute Physiologic and Chronic Health Evaluation (APACHE) II scores; in addition, KIM-1 quartiles were shown to correlate with dialysis requirement and hospital mortality [38]. A more recent report in the pediatric literature has described a 252 patient cohort, 7.1% of whom had AKI, in which KIM-1 levels measured in the emergency department had an area under ROC curve to predict AKI (pRIFLE criteria I) of 0.73 [34].

A recent study has explored urinary biomarkers in renal transplant recipients with worsening kidney function. In this study of 63 transplant recipients biopsied for worsening of kidney function, the authors reported that the rate of renal function decline over an average of 39.7 months was significantly correlated with urinary KIM-1 expression (but not with NGAL or IL-18). In addition, separating the patients into low and high KIM-1 groups, graft survival was significantly worse in the group with high KIM-1 expression [39]. 

### 2.4. Cystatin C

Cystatin C, a 13 kD protein of the cystatin family of protease inhibitors, is produced by all nucleated cells [73]. Serum levels of cystatin C have been established as a reliable correlate of GFR, superior to serum creatinine in that cystatin C production is not influenced by muscle mass; its level is not affected by age, race, or gender; and its urinary clearance does not involve tubular secretion. In addition, serum cystatin C appears to rise 1-2 days earlier than serum creatinine in the setting of AKI [74]. However, while serum cystatin C level can be used as a surrogate marker of GFR, it is not a true biomarker of AKI in that its levels are not a direct marker of renal injury.

Elevation in urinary cystatin C, on the other hand, is closer to a true biomarker of AKI. Cystatin C is freely filtered at the glomerulus and then nearly completely reabsorbed by the proximal tubules. Thus, any process that damages the renal tubules can impair cystatin C reabsorption, which means that AKI can manifest with elevated urinary cystatin C levels [75]. Specifically, it appears that elevated urinary cystatin C reflects renal tubular dysfunction, as opposed to glomerular injury [76].

Recent human studies on urinary cystatin C have shown promise in using this measurement as a biomarker of AKI, with cystatin C levels assayed by ELISA. In the setting of adult cardiac surgery, a study of 72 patients (34 with AKI) was used to construct ROC curves for urinary cystatin C that had AUC's of 0.705 for the immediate postoperative timepoint and 0.704 for the 6 hour postoperative timepoint [29]. Within three days of surgery, the ratio of urinary cystatin C to urine creatinine ratios had increased >20-fold in patients with AKI, compared to a 5-fold increase in non-AKI patients. In a study of 444 ICU patients (198 with AKI), urinary cystatin C had an AUC of 0.70 for the diagnosis of AKI and was found to be independently associated with sepsis, AKI, and death [30]. A study of 91 patients who received deceased-donor kidney transplants showed that urinary cystatin C could predict delayed graft function (AUC of 0.74 for the 6 hour urine cystatin C/creatinine ratio). In addition, the urine cystatin C/creatinine ratio on the first postoperative day was significantly associated with 3-month graft function [40]. 

## 3. Overview of Biomarkers in Toxic AKI

As detailed above, biomarkers of AKI have been studied extensively in the setting of ischemic AKI, both experimentally and in clinical scenarios in which ischemia is common (e.g., sepsis, cardiopulmonary bypass, etc.). In addition, there has been a considerable amount of research investigating biomarkers in nephrotoxic AKI. This area is of particular importance not only because toxin-mediated AKI is so common, but also because it may aid in the evaluation of drug safety in the future. In this section, we will consider the evidence for biomarkers in identifying toxin-mediated AKI.

### 3.1. Biomarkers in Contrast-Mediated AKI

Contrast-induced nephropathy (CIN), a form of AKI that results from the intravascular administration of iodinated contrast media for radiographic procedures, is a prominent cause of hospital-acquired AKI. CIN is thought to be the result of renal ischemia and vasoconstriction induced by hyperosmolar contrast media [77], and it manifests with a rise in serum creatinine typically within 2-3 days of contrast administration [78]. Although the standard definitions of CIN are based on a rise in creatinine over a few days, it is likely that the renal injury from contrast media begins immediately after administration and that sensitive early biomarkers could detect the injury sooner.

A study in 2006 examined urine and serum NGAL levels after percutaneous coronary intervention (PCI) in 35 adults [42]. None of these patients developed CIN, but significant increases in serum and urinary NGAL levels were found after PCI (<50% increases in both serum and urine NGAL at 4-hours postprocedure). A later study in 91 children undergoing cardiac catheterization and angiography found that in the 12% of patients who developed CIN, plasma and urine NGAL levels were excellent predictors of AKI within 2 hours of contrast administration (within 2 hours of contrast administration, urine NGAL was >10-fold higher, and serum NGAL was >4-fold higher in the CIN group than the non-CIN group) [41]. With a level of 100 ng/mL set as a cutoff, urine NGAL at two hours after procedure had a sensitivity, specificity, and ROC curve AUC of 73%, 100%, and 0.92, respectively. Plasma NGAL had similarly impressive test characteristics—sensitivity 73%, specificity 100%, and AUC 0.92. A subsequent study of 30 adults undergoing PCI reported that using a cystatin C-based definition of AKI, a 25% rise in serum NGAL had a sensitivity of 91.6% in diagnosing AKI, a specificity of 83.3%, and a positive predictive value of 95.6% [79]. A recent meta-analysis of studies involving NGAL in contrast nephropathy has reported that the AUC-ROC after contrast administration was 0.894 [43].

IL-18, described above as a powerful biomarker of ischemic AKI, has also been studied in the setting of CIN. A nested case-control study of 157 patients undergoing elective PCI did not identify significant differences between urine IL-18 levels in the 9.5% of patients who developed AKI and those who did not [44]. Conflicting results were reported in another study that included NGAL in its biomarker analysis. This study of adults undergoing PCI compared 13 patients who developed CIN with 27 controls. ROC curves were created for urinary IL-18 and NGAL measured at 24 hours, and the AUC's were found to be 74.9% and 73.4%, respectively [45]. Additionally, an increase of 25% from baseline in the biomarker level at 24 hours had an odds ratio for CIN of 10.7 for IL-18 and 5.0 for NGAL. Urinary IL-18 was an earlier predictor of CIN than serum creatinine, and, unlike creatinine, urinary IL-18 was found to be an independent predictor of later major cardiac events.

### 3.2. Biomarkers in Cisplatin Toxicity

Cisplatin is a major part of various solid tumor chemotherapy regimens, but significant nephrotoxicity is a major dose-limiting side-effect in up to 20% of patients receiving the drug. While most antineoplastic agents that act by crosslinking DNA cause damage exclusively to rapidly proliferating cells, cisplatin can also cause considerable damage to the relatively quiescent cells in the renal proximal tubule. This toxicity appears to be the result of local cisplatin accumulation within the proximal tubular epithelium, intracellular conversion of the drug to toxic metabolites, and resultant damage through multiple pathways [80]. Efforts to prevent the predictable nephrotoxicity have been hindered by the lag in diagnosing AKI by creatinine criteria, so there is great interest in identifying earlier and more reliable biomarkers of cisplatin-induced AKI.

Animal studies have demonstrated rapid induction of NGAL in proximal tubule epithelium within three hours of high-dose cisplatin administration. In addition, NGAL was readily detectable in the urine within three hours of dosing, compared to the 96-hour delay in serum creatinine changes [46]. Two other animal studies have reported that elevated levels of urinary KIM-1 can identify cisplatin-AKI earlier than plasma creatinine or blood urea nitrogen [48, 70], and a recent animal study has reported urinary cystatin C as a reliable marker of cisplatin-mediated AKI as well [49]. In addition to these well-established biomarkers, new biomarkers for cisplatin-mediated AKI have recently been reported (*α*-glutathione-S-transferase for proximal tubular injury, *μ*-glutathione-S-transferase for distal tubular injury, renal papillary antigen-1 for collecting duct injury, and clusterin for general kidney injury) [81]. 

There has been a recent report of using NGAL to identify cisplatin-induced AKI in humans. In this 2010 study, 24 patients who received cisplatin were studied, comparing 12 patients with clinically apparent AKI to those with stable serum creatinine levels. Serum and urine NGAL levels were measured at 1 and 4 hours after infusion as well as on days 1, 2, 3, 7, and 15 after infusion. Urine NGAL was found to significantly increase in the AKI group (a 1000% increase at day 1), and the rise preceded the creatinine-based diagnosis of AKI by 4.5 days. In addition, amongst the AKI patients, higher urine NGAL levels appeared to predict residual kidney dysfunction at 15 days [50]. 

Finally, urinary L-type fatty acid-binding protein (L-FABP), an emerging biomarker for AKI, was shown to increase within two hours of cisplatin administration, and L-FABP levels correlated with histological injury score and GFR earlier and more consistently than BUN measurements [51]. 

### 3.3. Other Nephrotoxin-Mediated AKI

Other forms of toxin-induced AKI have been studied, but there is less data than there is for contrast-mediated and cisplatin-induced AKI. Most of the studies involve KIM-1. A rapid assay for detection for urinary KIM-1 levels is under development, and in, rats, it was able to identify AKI caused by cadmium and gentamicin [71]. Other animal studies of urinary KIM-1 in gentamicin-, thioacetamide-, and cyclosporine-related AKI demonstrated that this assessment is a sensitive, specific, and accurate predictor of these drug toxicities [82]. KIM-1 has also been shown in animals to be an earlier marker for cadmium-induced nephrotoxicity than standard serum creatinine and urine protein measurements [83]. In addition, KIM-1 has also shown to increase in a dose-dependent fashion when rats are exposed to a variety of other nephrotoxins (hexachloro-1 : 3-butadiene, potassium dichromate, and cephaloridine [84].

Other biomarkers have been evaluated in drug toxicities, but not as extensively as KIM-1. One recent study examined twelve biomarkers in rats exposed to puromycin aminonucleoside (a glomerular toxin), finding significant NGAL increases after the toxin was administered [85]. Another separate series of animal experiments using a variety of medications with nephrotoxic side-effects (gentamicin, puromycin, vancomycin, cisplatin, doxorubicin, furosemide, tacrolimus, and lithium carbonate) showed promising results with a series of biomarkers including urinary levels of clusterin, cystatin C, total protein, and beta-2 microglobulin [86]. In humans, a 2009 study reported promising results using urinary cystatin C for monitoring tenofovir nephrotoxicity [87]. 

### 3.4. Metabolomic Profiling in Nephrotoxin-Mediated AKI

Metabolomics refers to the study of the metabolite pool within a cell, tissue, or biofluid under a particular set of conditions [88]. The field of metabolomics uses analytical techniques such as nuclear magnetic resonance (NMR) spectroscopy and mass spectrometry (MS) to identify and quantify a sample's metabolite pool. Not only can quantified metabolites serve as biomarkers of certain cellular or tissue injuries, they may also help elucidate mechanisms of injury, particularly in cases of drug toxicity.

Several animal studies have examined metabolomics in drug-induced AKI. For example, in rats treated with cisplatin for 48 hours, alterations in urinary glucose, amino acids, and Krebs cycle intermediates were observed, and changes in these metabolites preceded any changes in serum creatinine levels [89]. Another recent study in which rats were dosed with gentamicin, cisplatin, or tobramycin used a metabolomic analysis to demonstrate that increases in urinary concentrations of amino acids and polyamines could be detected after the first dose of a particular nephrotoxin, before any histopathological changes had occurred [14]. With prolonged exposure to these toxins, progressive urinary losses of amino acids with concomitant decreases of amino acid and nucleoside concentrations in kidney tissue were observed. Urinary concentrations of branched amino acids distinguished neurotoxin-treated samples from vehicle controls with 70%, 93%, and 100% accuracy after 1, 5 and 28 days of treatment, respectively.

### 3.5. Metabolomics in Calcineurin Inhibitors (CNI)-Induced Nephrotoxicity

One of the causes of both AKI and chronic kidney disease in renal transplant recipients is the use of CNI's [90], which are potent vasoconstrictors that have direct negative effects on the kidney. Unfortunately, CNI-associated chronic nephrotoxicity increases with duration of exposure and is often not fully reversible. This particular type of injury has been studied extensively within the field of AKI metabolomics.

A variety of studies have shown that CNI toxicity can manifest with qualitative changes in urine metabolite patterns that are typical for proximal tubular injury [10, 11, 91]. More recently, a series of studies has been published that involved the first attempts at metabolite quantification with CNI toxicity. These studies have identified CNI-induced changes in glomerular filtration, tubular secretion and absorption, and kidney cell metabolism, culminating in a proposal for a combinatorial metabolite marker for monitoring CNI-induced AKI [92]. This model involves markers of glomerular filtration (creatinine), reabsorption (glucose), tubular cell metabolism (citrate, oxoglutarate, and lactate), tubular secretion and kidney amino acylase activity (hippurate), and oxidative stress (isoprostanes). The combination panel also included the levels of trimethylamine-N-oxide, a metabolite that is released and protects against the protein-precipitating effect of uric acid. 

The association between metabolic changes induced by CNI's and the metabolites seen in urine has been confirmed by further proteomics studies in rats [93]. Compared to untreated controls, rats treated with CNI's showed changes in the expression of certain enzymes that explained several of the metabolite changes observed in urine. After six days of CNI treatment, rats showed urine metabolite patterns similar to those reported for agents known to cause oxidative damage, while pattern changes after 28 days were more typical of agents that cause S3 tubular damage [93, 94]. In addition, histological results showed evidence of proximal tubular damage. In aggregate, these studies suggest the following mechanism causing the characteristic changes in urine metabolite patterns: CNI's cause endothelial dysfunction and thereby derail mitochondrial oxidation, causing oxygen radical formation, inhibition of the Krebs cycle, and decline of energy production. Proximal tubular cells try to compensate by activating anaerobic glycolysis and importing Krebs cycle intermediates from urine.

Translation of the proposed combinatorial biomarkers into the clinical practice still has to be evaluated. However, there are encouraging reports that some of these metabolic changes have been seen in transplant patient populations [95–97]. In addition, there has been a report of a direct metabolomic evaluation of a particular drug in humans. In an open label, placebocontrolled, and crossover study, the time-dependent toxicodynamic effects of a single dose of oral cyclosporine A (5 mg/kg) on the kidney was assessed in thirteen healthy individuals [98]. The increase in urinary 15-F2t-isoprostane concentrations observed 4-hours after the administration of cyclosporine indicated an increase in oxidative stress. Unbiased metabolome PCA analysis revealed significant changes in urine metabolites typically associated with negative effects on proximal tubular cells. The major metabolites that differed between the 4-hour urine samples after cyclosporine and the placebo were citrate, hippurate, lactate, TMAO, creatinine, and phenylalanine. Creatinine concentrations in serum remained unchanged. A decrease in citrate concentrations in urine kidney transplant patients had also been reported by others [99]. These results indicate that analysis of urinary metabolites was a sensitive enough maker for the detection of the effects of a single cyclosporine dose already shortly after drug administration. These results also suggested that previous results in rats may translate into humans.

## 4. Conclusions

Serum creatinine was first used for the determination of kidney function around 1917 [100]. Nearly, 100 years later, serum creatinine is still the major determinant of kidney function. The diagnosis of AKI has traditionally relied on serum creatinine, a lab measurement fraught with problems. Difficulty in identifying AKI quickly and accurately has led to significant problems in patient care, drug development, and nephrotoxicity monitoring. New biomarkers of AKI have been studied in a variety of animal studies and clinical settings and show promise for facilitating earlier and more accurate recognition of AKI. Further research is needed to understand the performance of these tests in the settings of other types of renal injury, but as the use of biomarkers become more widespread and accepted, our ability to identify nephrotoxic insults and care for patients with impaired kidney function should improve.

## Figures and Tables

**Table 1:** Studies of AKI Biomarkers.

Clinical setting	Patient population	Biomarker	References
cardiac surgery	pediatric	NGAL	[16, 17]
		IL-18	[18]
		NGAL + IL-18	[19]
	adults	NGAL	[20–24]
		IL-18	[25, 26]
		NGAL + IL-18	[19]
		KIM-1	[27, 28]
		urinary CysC	[29, 30]

critical care	pediatric	NGAL	[31, 32]
		IL-18	[33]
		KIM-1	[34]
	adults	NGAL	[35–37]
		KIM-1	[38]

post-transplant	adults	KIM-1	[39]
		urinary CysC	[40]

contrast-induced AKI	pediatric	NGAL	[41]
	adults	NGAL	[42, 43]
		IL-18	[44]
		NGAL + IL-18	[45]

cisplatin-induced AKI	animal studies	NGAL	[46]
		KIM-1	[47, 48]
		urinary CysC	[49]
	adults	NGAL	[50]
		L-FABP	[51]
